# Reflective analysis on empirical theories in consciousness

**DOI:** 10.3389/fpsyg.2025.1571098

**Published:** 2025-09-25

**Authors:** Hongju Pae

**Affiliations:** Department of Cognitive Sciences, University of California, Irvine, Irvine, CA, United States

**Keywords:** consciousness, embodied cognition, phenomenology, subjectivity, theories in consciousness

## Abstract

Contemporary theories of consciousness offer a range of explanatory perspectives. Global Workspace Theory emphasizes cognitive access, Higher-Order Theories focus on metacognitive representation, Integrated Information Theory centers on intrinsic experience, and Predictive Coding Theory models cognitive processes as probabilistic inference. While each theory provides valuable insights, they often remain in conflict due to differing assumptions about the nature of consciousness. This paper proposes a phenomenologically informed framework that clarifies the explanatory scope of each theory in relation to key features of lived experience. Rather than seeking to reduce consciousness to a single principle, I argue for a pluralistic approach that respects the distinctive contributions of each model. Through comparative analysis guided by phenomenological reflection and supported by recent interdisciplinary proposals, I show how these theories can be seen as addressing complementary dimensions of consciousness. The aim is not to construct a single unified theory, but to demonstrate how integration, grounded in reflective phenomenological analysis, can serve as a starting point toward a more adequate science of consciousness.

## 1 Introduction

What does it mean to be conscious? What constitutes a lived experience, and what gives us the sense that we exist here and now, as ourselves? These enduring questions traverse the philosophy of mind, neuroscience, and cognitive science – yet a definitive answer remains elusive. This manuscript begins from a concern that current debates in consciousness studies often talk past each other – not because any single theory is wholly wrong, but because each targets a different explanatory domain under the umbrella term ‘consciousness'. Rather than choosing sides, this paper argues for a pluralist perspective: one that treats theoretical divergence not as conflict, but as an opportunity for conceptual integration.

To pursue such integration meaningfully, we must first clarify what each theory is actually trying to explain. Phenomenology, often understood as reflective analysis, is used here as a clarifying method for determining what each theory of consciousness is really trying to explain. In contrast to conceptual classification, phenomenological analysis brackets preconceptions to illuminate the structural conditions under which conscious experience becomes meaningful (Embree, 2011). That being said, this paper does not suggest that theoretical integration will yield an immediate resolution to all challenges in consciousness studies. Rather, it aims to move beyond rigid polarization by fostering a more collaborative and integrative discourse, one in which diverse theories can mutually inform and enrich one another within a conceptually clarified space. Each theory, in its own way, captures something essential. The challenge—and the opportunity—is to consider how these partial insights might be integrated, not through reduction or assimilation, but through careful articulation of their respective contributions.

While grounded in phenomenological reflection, this inquiry is not pursued in isolation from scientific discourse. Recent decades have witnessed the emergence of several influential empirical theories of consciousness—Global Workspace Theory, Higher-Order Theory, Integrated Information Theory, and Predictive Coding Theory—each offering distinct explanatory targets and methodological commitments. Yet despite their empirical sophistication, these models often begin with assumptions that diverge from phenomenological insights. Some emphasize functional accessibility, others intrinsicality, still others inferential dynamics. What is often missing, however, is sustained reflection on the structural features of conscious mental phenomena as such: its intentional orientation, its temporal unfolding, its embodied situatedness, and its pre-reflective self-acquaintance. A phenomenological methodology, while not a replacement for scientific theory, can help clarify what is at stake in such models by specifying the explanandum with greater precision. This paper thus aims to place phenomenological reflection in conversation with contemporary scientific theories, not to adjudicate between them, but to illuminate the deeper structural questions they raise, and to map how their insights might be productively combined.

Accordingly, the remainder of this manuscript is structured as follows: Section 2 provides a philosophical groundwork by clarifying the methodological tensions between representationalist assumptions and phenomenological approaches to consciousness. Section 3 introduces four core structural features of consciousness derived from reflective phenomenological analysis. Section 4 examines four major scientific theories of consciousness—Global Workspace Theory, Higher-Order Theory, Integrated Information Theory, and Predictive Coding Theory—and assesses their correspondence with the phenomenological features discussed in Section 3. Section 5 presents a unified framework that maps each theory onto different dimensions of conscious experience and explores how these theories might be integrated rather than treated as competitors. Finally, Section 6 offers concluding reflections on the philosophical role of phenomenology in grounding interdisciplinary theories of consciousness.

Throughout this paper, several terms such as intentionality, qualia, awareness, attention, and self-awareness are used in their phenomenological sense. *Intentionality* refers to the structural directedness of consciousness toward an object or meaning, not merely to representational content. *Attention* refers to the selective modulation or amplification of particular aspects within experience, which determines what becomes prominent, but is not a prerequisite for intentionality. *Qualia* denotes the pre-reflective “what-it-is-likeness” of experience, rather than atomistic or intrinsic mental features. *Awareness* is used as a general term that encompasses both self-awareness and world-awareness, that is, our awareness of objects, situations, and ourselves. Within the former category, a key distinction is made between *reflective self-awareness* and *pre-reflective self-awareness*. Reflective self-awareness involves a thematic or introspective recognition of oneself as a subject, while pre-reflective self-awareness refers to the minimal, tacit sense of mine-ness that accompanies all experience. These distinctions are crucial for avoiding conceptual conflations and follow the phenomenological tradition rather than analytic usage.

Also, while this paper draws on conceptual features from Husserlian phenomenology—such as intentional matter and quality, or pre-reflective self-consciousness—note that it does not adhere rigidly to the full transcendental framework of early phenomenology. Instead, these concepts are used heuristically as structural tools for analyzing conscious experience. In particular, they are re-situated within a broader framework that integrates phenomenological reflection with embodied and enactive approaches. The aim is not to offer a doctrinal reading of classical phenomenology, but to adapt its insights for interdisciplinary engagement. The terminology and distinctions developed here are shaped to minimize conceptual ambiguity and to support theoretical integration across philosophical and scientific domains.

## 2 Investigating the science of consciousness through phenomenology

### 2.1 Historical background and the epistemological divide

A principal tenet of phenomenological inquiry is that the structure of phenomena, as they appear to one, is essentially the same as the structure of phenomena as they appear to another. This statement may seem ordinary at first glance, but it stands in contrast to what is often taken as the dominant approach within analytic philosophy which conceives of mental content as mediated through representational structures. On this representationalist view, cognition involves internal representations that stand in for external states of affairs, with mental states typically treated as distinct from the world they represent.^1^ This epistemological stance nonetheless diverges significantly from phenomenological thinking, which denies that consciousness consists in the internal mirroring of an external world and instead treats the world as disclosed within lived experience. Modern cognitive science and psychology, largely rooted in this dominant representationalist tradition, are shaped by a model of animal-environment dualism in which the mind is internal and the world is external. It is thus unsurprising that the naturalistic approach to consciousness tends to adopt a third-person point of view. The familiar formulations of the Mind–Body Problem, the so-called Hard Problem, and the explanatory gap all inherit this framework, which presupposes a division between mental experience and physical reality. Consciousness, especially in its first-person and subjective dimensions, resists full objectification within this paradigm, and it is precisely this resistance that gives rise to the enduring difficulty of the explanatory gap and the persistence of the Hard Problem.^2^

However, we must not be misled by this inherited dichotomy. To sharply distinguish the so-called Hard Problem from the Easy Problems reflects an underlying assumption that selected functional or cognitive aspects of consciousness suffice to explain the whole. While reductionist frameworks often present themselves as offering efficient solutions to complex problems, such explanatory efficiency may come at the cost of fidelity to the phenomenon itself. If the proposed explanatory link misrepresents the explanandum, or if the explanandum is itself inherently irreducible, then the explanation fails in principle. Moreover, classifying consciousness into categories such as Phenomenal consciousness or Access consciousness, as suggested by Block (1995), risks oversimplifying the mental processes and leaving their intrinsic structure unaccounted for. This assumption is not incidental, but deeply rooted in the history of cognitive science itself. Early theorists such as Fodor (1975) and Gardner (2011) conceptualized the mind as a computational system that processes internal symbolic representations in response to external stimuli, thereby entrenching a dualistic framework that separates the internal subject from the external world. On this view, consciousness becomes epistemically indirect, reducible to discrete internal states governed by syntactic operations.

In response to this legacy, phenomenologists and advocates of embodied cognition have argued that such reductionist views fail not only by leaving out key experiential aspects, but more fundamentally by misconstruing the ontological relation between consciousness and world. Varela et al. (1991), for instance, argue that experience is not a representational mirroring of a world external to the subject, but a situated enactment. Thompson (2007) extends this view by showing that consciousness is inseparable from the biological and experiential conditions of the organism. Zahavi (2005) similarly emphasizes that consciousness is not an internal mirror of an external world, but the condition under which the world is disclosed as meaningful. On this view, the world is not encountered as a domain external to the subject and waiting to be represented, but is instead disclosed within the subject's lived, situated engagement. Hence, consciousness is not to be understood as a container of inner mental states, but rather as the very locus in which the world becomes meaningful.^3^

### 2.2 The misconception of introspection

Phenomenology is often mistakenly equated with introspection, yet the two differ in significant ways. Introspection typically assumes a division between inner mental content and the external world, examining internal states from a quasi-observational stance. Phenomenology, by contrast, seeks to bracket such preconceptions and to illuminate how consciousness and world co-constitute one another through lived experience. In fact, classical phenomenologists such as Merleau-Ponty and Gurwitsch have emphasized that phenomenology is not merely a descriptive report of inner life, but a systematic method for disclosing the structural conditions of meaning and appearance (Merleau-Ponty, 2013, p. 201–216; Gurwitsch, 2010, p. 89–106).

That said, the line between phenomenological reflection and introspection is not always rigid. Some phenomenologists acknowledge similarities in first-person focus or experiential orientation, even while insisting on key methodological differences. However, whereas introspection may treat inner states as objects of observation, phenomenology discloses the structures by which experience itself is constituted. The emphasis is not on cataloging mental contents, but on uncovering the intentional and contextual conditions through which meaning becomes available. Thus, while phenomenology and introspection may share a concern with first-person experience, they are distinguished by their aims and philosophical commitments. Phenomenology does not claim epistemic immediacy, but instead calls for a methodologically disciplined suspension of naturalistic assumptions. Its aim is to clarify the very conditions under which phenomena are given, not merely to describe their occurrence. This distinction, while subtle, is central to the phenomenological method.^4^

### 2.3 On intentionality

Phenomenology identifies intentionality as one of the most universal and crucial characteristics of consciousness. More precisely, phenomenology most clearly distinguishes itself from analytic philosophy in its treatment of intentionality. Brentano, who first introduced the term into phenomenology, defined intentionality as the most decisive feature that distinguishes conscious mental processes from non-conscious processes (Brentano, 2014, p. 68–69).^5^ Acknowledging the existence of intentionality as an irreducible attribute and investigating it from the standpoint of the first-person subject grants phenomenology its unique specificity. A common misconception is that phenomenal and qualitative aspects of mental processes are non-intentional because they are not acts of directed representation. This construal risks reducing intentionality to a mechanism for reportable or functionally accessible mental content, that is, to something akin to what is often called Access consciousness.

However, in phenomenology, intentionality encompasses not just the object of experience but the particular mode in which the world is disclosed. Conscious experience is not merely directed at an object; it is a specific way in which a subject encounters the world, making phenomenality about something (world-representing) and about someone (self-involving) (Sartre, 2021, p. 3–5). The world is not only manifested to the subject but also disclosed as meaningful through the subject's embodied and contextual stance toward it. This is not merely a difference in representational content or mode, as often acknowledged in analytic theories, but a deeper structural distinction: in phenomenology, intentionality is the mode of disclosure itself – not a relation to an object, but the condition under which something can appear as meaningful in the first place.^6^

Most importantly, according to Husserl, the intentional object (intentional matter) and the act of consciousness (intentional quality) are not two separable entities but are essentially co-constitutive (Husserl, 2001, Vol. I, p. 193–194; Vol. II, p.119–120). This implies that a purely third-person perspective on consciousness, one that excludes its lived, first-person givenness, cannot be regarded as ontologically neutral. Rather, it must be understood as already shaped by a specific intentional stance. In this light, the idea of complete objectivity does not refer to an absolute or independent fact, but to an idealization that emerges within a particular framework of epistemic commitments.

### 2.4 Embodiment and the mind-world relation

The perspective that treats mind and world not as separable entities but as dynamically intertwined naturally extends to the relationship between mind and body. While Husserl (1989, p. 57) laid the groundwork for phenomenological reflection on the body through concepts such as the *lived body (Leib)*, the full articulation of embodiment as constitutive of cognition emerges more forcefully in the work of Merleau-Ponty and later enactive accounts of mind (Merleau-Ponty, 2013, p. 76, 350–351; Varela et al., 1991; Clark, 1997; Thompson, 2007). The world is disclosed to us through bodily experience, such that reflection on how the world is experienced becomes unintelligible in the absence of physical self-awareness. This orientation permits an evolutionary interpretation of consciousness, not as an accidental byproduct, but as something shaped by environmental adaptability and the biological viability of embodied forms (Varela et al., 1991).

However, this should not be misunderstood as a naturalization of the mind in the manner of biological reductionism. The aim is not to explain consciousness merely as a functional product articulated in the vocabulary of neuroscience. Rather, the very fact that the mind is embodied explains the interdependency of mind and world. Through our lived body, we encounter the world as embodied subjects. For this reason, phenomenology calls for a clear distinction between the *lived body (Leib)* and the *objective body (Körper)*, and insists that only the former captures what is essential in consciousness. Here, the objective body is the body as observed from a third-person perspective, as a physical object among others. The lived body, by contrast, is the body as it is experienced from within, as the medium through which the world is disclosed and acted upon (Merleau-Ponty, 2013, p. 329–330). This conceptual distinction highlights phenomenology's resistance to biological reductionism, treating embodiment not as a physical substrate but as the existential basis of lived experience. Hence, phenomenology does not equate the mind-world relation with that of mind and brain. To do so would be to adopt the naturalistic paradigm that phenomenology explicitly aims to critique. Just as it resists the assumptions underlying the Mind-Body Problem, it also challenges the framing of the so-called Hard Problem.

To summarize, phenomenology understands the mind not as something set apart from the world, but as a mode of being in and toward the world. Its central effort lies in clarifying this relation through the concept of intentionality. In exploring consciousness, phenomenology treats the oppositions between subjectivity and objectivity, first-person and third-person perspectives, and mind and body not as absolute dichotomies, but as provisional constructs that conceal the continuity of experience. Such dualisms are themselves intentional attitudes that have not been bracketed, which obscure the essence of consciousness by placing conceptual filters over its givenness. Phenomenology, as a mode of *reflective analysis*, aims to restore this givenness. In seeking to make consciousness amenable to scientific inquiry, it insists that the explanandum must be preserved in its integrity. By engaging in rigorous reflective analysis, phenomenology offers a method for keeping consciousness as it appears, and for understanding its structure from within.

## 3 Properties of consciousness discovered by reflective analysis

Before proceeding, it is imperative to clarify a methodological point: the characteristics of consciousness must not be predefined in advance. Phenomenologically speaking, what holds ultimate authority is the phenomenon as it appears to the subject. Paradoxically, even well-intentioned efforts to clarify the structure of consciousness through theoretical categorizations often risk obscuring what they seek to illuminate. Categories such as Creature consciousness, State consciousness, Phenomenal consciousness, and Access consciousness may be heuristically useful, but they are not phenomenologically primary. From the standpoint of reflective analysis, it is precisely such assumptions that must be bracketed at the outset.

Accordingly, the features introduced in this section are not intended to serve as necessary or sufficient conditions for consciousness. To pursue such definitional aims would not only violate the spirit of phenomenological inquiry but would also diverge from the purpose of this paper. The aim here is more modest and exploratory: to outline several structural features that typically accompany conscious experience, and to consider how these may be disclosed through eidetic variation and reflection. These features are offered not as rigid criteria, but as provisional signposts, or conceptual tools, to aid inquiry into what makes consciousness the phenomenon that it is. At the same time, consciousness must be approached as a totality within which these features are always already intertwined. The analytical distinctions that follow serve only a heuristic function. They are not meant to suggest that consciousness can be decomposed into isolated parts or divided into mutually exclusive domains.

While some may perceive a tension between describing consciousness in essential and universal terms on the one hand, and emphasizing its contextual, embodied, or evolutionary dimensions on the other, this paper does not regard this as a contradiction. The essential features of consciousness are not abstracted from lived reality but are always instantiated through concrete experience. They are repeatable structures that manifest within variation, not in spite of it. Merleau-Ponty suggests that what appears as a priori is not imposed externally or abstracted from experience, but arises immanently through the structure of lived events themselves (Merleau-Ponty, 2013, p. xii–xiii). In this sense, phenomenological generality is not pre-given in a rigid form but unfolds within the variability of concrete experience. It is through this layered understanding, where generality and particularity are seen as mutually informing, that the following analysis proceeds.

### 3.1 Pre-reflective self-acquaintance

The primary givenness of self-acquaintance is widely recognized in phenomenological discourse as a constitutive feature of consciousness. Heidegger holds that in every encounter with the world, *being-in-the-world (Dasein)* is not merely present but disclosed as essentially *mine (Jemeinigkeit)*, such that selfhood is not derived from reflection but is always already a constitutive structure of being-in-the-world (Heidegger, 1996, p. 114–117). Sartre articulates this insight most directly, arguing that pre-reflective self-consciousness is not an additional act but the very mode of being of consciousness itself: “We should not regard this consciousness (of) self as a new [act of] consciousness but as the only possible mode of existence which is possible for a consciousness of something” (Sartre, 2021, p. 13).^7^ Self-acquaintance is therefore not an auxiliary property but a fundamental condition that makes intentionality itself possible. Consciousness is not constituted by an act of self-reference; rather, self-referentiality is intrinsic to what it means for something to be conscious.

Note that this paper does not assume that all conscious acts must be directed toward an object in the narrow Brentanian sense. In particular, pre-reflective self-awareness—the immediate awareness we have of ourselves as experiencing—is not structured as an act that presents an object. Yet this does not place it outside the structure of consciousness. Rather, it functions as a foundational condition for intentionality itself. It does not possess intentionality in the standard object-directed sense, but provides the existential ground from which all intentional orientation becomes possible. In this respect, consciousness can be said to presuppose a minimal self-recognition that is not directed but is nevertheless constitutive of all directedness.

This pre-reflective self-awareness underlies the privacy of conscious experience, marking first-person subjectivity as phenomenologically distinct from any third-personal stance. Since every conscious experience is intrinsically “mine,” the subject cannot stand apart from their own consciousness as though it were an external object. This makes it impossible, in principle, to experience another's consciousness as one's own, reinforcing the idea that conscious experience is irreducibly first-personal. Without this self-acquaintance, no mental state could be experienced as present, rendering it phenomenologically non-conscious (Kriegel, 2009).^8^ From this perspective, the ineffable and irreducible qualitative aspects of consciousness, what is often called *qualia*, must also be understood through the lens of self-acquaintance. The impossibility of experiencing “what it is like to be another entity without being that entity” underscores that consciousness is always attributed to a singular self-aware existence.

Objections may arise from altered states of consciousness, such as hallucinations or near-death experiences, which appear to disrupt the coherence of self-awareness. Yet these cases do not negate the structure of self-acquaintance but rather reveal its flexibility under novel conditions of experience. Sartre's account of implicit self-awareness suggests that even in radically altered states, a minimal self-reference persists (Sartre, 2021, p. 1–7). This view is echoed in Zahavi (2005, p. 133–146), who similarly argues that even in disrupted or pathological forms of consciousness, a minimal structure of mineness remains operative. These accounts converge on the idea that self-awareness is not a secondary achievement, but a structural feature of consciousness that remains even when the coherence of experience is compromised. Hence, such altered states or pathological cases may instead be understood as breakdowns in the horizonality of experience, that is, interruptions in the habitual synthesis between self and world, not eliminations of the first-personal structure itself.

In summary, our experience consistently exhibits a basic form of self-givenness, even in moments where the sense of self appears diminished or unstable. Whether articulated in terms of Heidegger's notion of mineness *(Jemeinigkeit)*, Sartre and Zahavi's pre-reflective self-awareness, or Merleau-Ponty's bodily intentionality, the conclusion remains consistent: consciousness is always, in some manner, given to itself. Any systematic account of consciousness must therefore begin not with a constructed ego or reflective self-concept, but with the immediate and non-thematized self-acquaintance that implicitly grounds all conscious experience.

### 3.2 Contextualized, environmentally situated

Phenomenological inquiry has long regarded intentionality as the structural core of consciousness, based on the idea that consciousness is always directed toward something. Although this claim serves as a foundational principle, it has been developed in varying ways across different strands of the tradition. In classical phenomenology, particularly in Husserl's transcendental framework, intentionality is understood as a correlation between acts of consciousness and the way in which the object is given or disclosed to the subject within experience, all structured within a broader horizon of meaning. Subsequent thinkers such as Merleau-Ponty and Sartre, along with contemporary phenomenologists influenced by enactivist approaches like Noë and Thompson, reinterpreted it through a more embodied, affective, and context-sensitive lens. This section adopts that latter view, focusing on intentionality not as a fixed relation but as a mode of orientation shaped by the subject's lived engagement with the world.^9^

Consciousness is never encountered in isolation. Every act of perception, cognition, or affect arises against a background shaped by bodily presence, environmental affordances, and socio-cultural patterns. Even direct perception is not a mere reaction to stimuli, but a gestalt-like integration within a meaningful whole (Merleau-Ponty, 1963, p. 129–136). On this basis, phenomenologists like Gallagher and Zahavi (2007, p. 94–104) argue that sensations are not the building blocks of perception but theoretical abstractions imposed on already structured experience. Classical illusions such as the Müller-Lyer lines and Ebbinghaus illusion illustrate that perception depends less on input than on contextual configuration. As Noë observes, “Phenomenology presents experience as something that could not occur in the absence of situations and things” (Noë, 2007, p. 235).

Importantly, it is intentionality that underlies and enables the contextual nature of conscious experience. Consciousness is not merely influenced by context; rather, it is structurally predisposed to be contextual because it is always directed toward the world. This directedness is not neutral or passive – it involves taking up a perspective. To intend something is to approach it from a particular perspective, shaped by one's personal history and environmental situation. This account of intentionality avoids a reductive or naïve interpretation that treats it as mere object-directedness. Instead, following Husserl's notion of horizon-intentionality and Merleau-Ponty's embodied “intentional arc,” we understand intentionality as the very structure through which context is constituted, not simply received (Husserl, 1973, Part II; Merleau-Ponty, 2013, Part I, Chapter 3). For instance, a reader approaching the same text either with sympathetic resonance or with skepticism will undergo a markedly different experience, not because the content of the text has changed, but because consciousness engages the text from a situated perspective. To borrow a concept from Husserl, it may be said that the intentional quality of consciousness accounts for this kind of contextualization (Husserl, 2001, Vol. II, p. 119–120). In this sense, intentionality is what binds experience to context and allows meaning to emerge from lived engagement rather than from passive reception.

Consciousness should thus be understood as a dynamically unfolding process, shaped by past experience, bodily orientation, affective stance, and social context. Hence, subjectivity is not enclosed but formed through ongoing relation to the world and to others. Intersubjectivity plays a particularly important role here, as other humans are not encountered as objects but as subjects who perceive and interpret as we do. Sartre calls this the “decentralization” of my world (Sartre, 2021, p. 307–331), through which the recognition of others' perspectives transforms and reconfigures my own. In this way, subjectivity is never entirely self-contained but is always situated among other subjectivities. Moreover, the ability of human beings to function as empathetic social beings may be seen as rooted in the fundamentally contextualized nature of consciousness itself. Scheler (2008), for instance, understands empathy as a direct, affectively informed intuition of recognition of the other, not one grounded in analogy or inferential reasoning. Similarly, Stein (1964) maintains that empathy is not a projection or imaginative act, but a mode of intentionality through which the other is disclosed as a subject of experience.

On this basis, consciousness cannot be understood as solipsistic or egocentric. A conscious individual is not a self-enclosed mind but a being situated with others in a shared world. The horizon within which experience unfolds is already intersubjective; first-person experience itself implicitly acknowledges the presence of other perspectives. Through this interpretation, the irreducibility and richness of lived experience are preserved without being fragmented into decontextualized elements.

### 3.3 Spatio-temporal expandability

Having established the contextual and environmentally situated nature of consciousness, I now turn to two phenomenologically significant dimensions that arise from this orientation. This section explores two frequently overlooked aspects of conscious experience—spatiality and temporality—both of which reveal the intrinsic openness and dynamic structure of consciousness.

Let us begin with spatiality. Our experience of the world is always mediated through the body, which is not merely perceived as an external object but is felt as an integral part of the self. This bodily embeddedness is coupled with a form of self-acquaintance that does not require reflective observation. In ordinary, non-pathological conditions, we do not perceive our bodies as belonging to someone else or as neutral objects in the world. Rather, we live through them as integral parts of ourselves. From a phenomenological standpoint, perception is not the passive registration of sensory data. It is an active engagement that includes bodily movement and orientation toward the environment (Gibson, 2014, Merleau-Ponty, 2013, Part I; Noë, 2004). Husserl emphasized that kinesthetic movement and sensory access are not two separate functions but aspects of a unified structure. In his view, action and perception co-constitute the experience of the world (Husserl, 1989, Section II, Chapter 3). This means that even in simple perception, such as seeing a red apple, we do not perceive it as a flat visual surface. Rather, we grasp its spatiality by anticipating its hidden sides, its volume, and its affordances. The back of the apple, though not visible, is still present in our perceptual grasp. This inference of spatial extension arises because perception is always embodied. Our movements, whether by turning the object or by shifting our viewpoint, reveal that we are situated agents. Through this bodily embeddedness, our experience expands across spatial horizons. As Noë (2004, p. 1) puts it, “What we perceive is determined by what we do or what we know how to do. It is determined by what we are ready to do. In ways I try to make precise, we enact our perceptual experience; we act it out.” Perception, then, is not merely visual appearance, but an active exploration made possible by the body's capacity to move, anticipate, and engage with the world.

This spatial openness also plays a role in how we experience others. When I recognize another person within the same physical space, I do not merely register a body. I encounter an agent, someone whose actions express an intentional perspective. To understand another person is to engage in a form of empathetic exploration, which is not unlike exploring the hidden side of an object. It involves socio-interactive probing, a kind of exploratory engagement that reveals the other's subjectivity. Merleau-Ponty and Sartre point out that our experience of others is structured by spatial interrelation and bodily responsiveness (Merleau-Ponty, 2013, Part II, Chapter 4; Sartre, 2021, Part III, Chapter 1). From this perspective, the social interaction involved in understanding another mind can itself be conceived as a form of spatial extension.

Let us now turn to temporality. Consciousness is not frozen in isolated moments. It unfolds across time and carries within it both memory and anticipation. Despite the discrete nature of neuronal firing, our conscious experience is surprisingly continuous. For example, the act of reading this text likely would not feel segmented or choppy – instead, it is continuous. Moreover, our sense of time is context-dependent. Our metaphorical language reflects this embedded temporality: time seems to slow down during hardship and to fly by in moments of joy. These expressions, though figurative, highlight that our experience of time is shaped by context, mood, and bodily state. Consciousness is temporally structured not by clock time but by lived significance. Husserl's analysis of time-consciousness highlights how each experience of the present is constituted by a primal impression, which is always accompanied by a retention of the immediate past and a protention of what is about to come (Husserl, 1991, p. 28–35). These structures—retention, primal impression, and protention—are not discrete acts but interwoven aspects of every conscious moment. Retention is not the same as remembering, and protention is not simply predicting. Rather, they are intrinsic features of temporal givenness. Just as our spatial field includes what is not currently seen but remains anticipated, our temporal field includes what is not yet present but still felt as imminently unfolding.^10^

Taken together, these insights suggest that consciousness is expansive across both spatial and temporal dimensions. However, this expansiveness is not limited to physical or chronological domains. Higher capacities such as abstract thought, imagination, and logical reasoning may be understood as further expansions of intentionality. After all, even applying phenomenological methodology to one's experience is utilizing such expandability. These acts do not occur outside space and time, but they depend upon the very expandability that bodily and temporal orientation make possible. Every act of consciousness, whether directed toward a physical object, another sentient individual, a memory, or a conceptual structure, presupposes a subject already embedded within a meaningful horizon. In this light, consciousness is not a mere assemblage of discrete factors but a unified experiential field. It unfolds across space, time, and the horizon of possibility.

### 3.4 Selective attention focus toward intended matter as an agent

Let us now consider the capacity for selective attention as an essential dimension of conscious agency. It may appear self-evident that consciousness of a particular intentional content requires attention to be directed toward that content. Yet, as discussed in Section 2, not all forms of consciousness involve explicit object-directedness.^11^ For instance, when reaching to grasp a mug, one would rarely be consciously attending to each muscular contraction of the fingers or to the proprioceptive adjustments that guide the fingers. Even when our attention is engaged elsewhere, we can perform such bodily actions with relative ease. Similarly, when walking or driving while speaking to someone, our bodily movements are carried out fluently, without requiring focused cognitive effort. The more familiar an action becomes, the less attentional demand it imposes, and the more the body itself recedes into transparency within our experience (Merleau-Ponty, 1963, Part II; Sartre, 2021, Part IV).^12^ Nonetheless, even in such cases, we retain an immediate awareness that it is ourselves who are performing certain actions – grasping the mug, walking through the space, or steering the car. This sense of agency is not derived through observation or inference but is directly given in the pre-reflective self-acquaintance that accompanies intentional action.

What, then, distinguishes mere movement from action? What renders a conscious subject an *agent*, rather than a passive locus of bodily motion? Consider the simple case of being displaced from point A to point B. There is a phenomenological distinction between walking from A to B with the purpose of arriving there and being pushed from A to B by an external force. Though the bodily displacement is the same, the experience of agency differs entirely. This distinction, foundational in the philosophy of action since Anscombe's analysis of intentional action as purposive movement (Anscombe, 1957), has also been explored from a phenomenological perspective by Gallagher and Zahavi. They approach agency not only in terms of intention but through the subject's first-person experience of initiating and controlling bodily movement. In their account, action is enacted through the lived body and structured by pre-reflective awareness, rather than defined merely by causal antecedents or external observation (Gallagher and Zahavi, 2007, p. 158–169).

One important insight is that action can be governed by the subject's own intention. In such cases, the content toward which intentionality is directed can often be selected or maintained by the subject. When a conscious state includes a sense of agency, the orientation of attention may align with the subject's guiding purpose. If we understand attention as a mode of directed action, then focusing attention on a particular object becomes itself a meaningful exercise of agency. Consider, for example, the experience of being interrupted by a sudden loud noise while reading. Phenomenologically, such a disruption resembles being physically pushed off one's intended course. This suggests that attentional focus is not fixed but may be disturbed, redirected, or actively restored. Attentional direction thus reveals itself as a structured and dynamic act.

Moreover, attention can function at multiple levels. At one level, it operates as a perceptual focus directed toward a given object. At another, it may take the form of awareness of one's own attentional state, often referred to as metacognitive awareness. In this latter case, the subject does not merely attend, but also reflects on the act of attending. From this perspective, metacognitive awareness may be interpreted as a higher-order act of attention that makes one's own orientation a possible object of scrutiny. The capacity to shift, regulate, and reorient such attentional structures according to one's ongoing aims or context constitutes an important form of agentive involvement. Thus, to be capable of directing the focus of attention purposefully is, in effect, to possess the ability to determine the structure of one's attention.

Nevertheless, phenomenology does not maintain that attentional focus is a necessary condition of all conscious states. The aim here is not to suggest that attention must be present for consciousness to occur, but rather to clarify that attention, when present, can be seen as an intentional act in its own right, or as an extension of agency. Not all forms of consciousness are intentional in the narrow sense. For instance, pre-reflective self-consciousness, as discussed earlier, is not object-directed, yet it is undeniably a constitutive feature of experience.

Even if attention is not an intrinsic property of all consciousness, it remains something that is actively guided by the subject. Insofar as it is purposive and directed, it carries intentional structure and may be understood as part of consciousness. When selective attention is grounded in a sense of agency, it becomes continuous with other forms of intentional action. As Sartre argues, consciousness manifests both in pre-reflective and reflective forms, with reflective consciousness always presupposing a more basic, pre-reflective awareness (Sartre, 2021, p. 153–155). Therefore, when a subject intentionally focuses attention on an object, that act not only discloses the object but also affirms the underlying structure of self-awareness through which such direction becomes possible.

## 4 Empirical theories of consciousness and its critiques

In this section, I examine how several influential scientific theories of consciousness attempt to account for the properties discussed in the preceding section. The four primary theories under consideration, as outlined by Seth and Bayne (2022), are: (1) Global Workspace Theory (GWT), (2) Higher-Order Thought Theory (HOT), (3) Integrated Information Theory (IIT), and (4) Predictive Coding Theory (PCT). While these approaches differ in their theoretical aims and explanatory scope, they generally prioritize either conscious accessibility (Access consciousness) or qualitative subjectivity (Phenomenal consciousness). Though this contrast is commonly invoked, it is not always clearly defined, and the distinction itself is contested within both analytic and phenomenological traditions. What unites these theories, however, is a tendency to explain one facet of consciousness in isolation, often under the assumption that doing so suffices to account for the phenomenon in its entirety. In what follows, I briefly outline the key claims of each theory and assess their scope and limitations from a phenomenological standpoint.

Before turning to the individual theories, it is important to clarify the broader philosophical orientation from which this critique proceeds. Contemporary models such as GWT and many versions of HOT often construe consciousness in functional or representational terms – either as reducible to informational content, or as dependent on metacognitive or higher-order access. These models typically adopt a third-person explanatory framework, privileging cognitive accessibility, behavioral reportability, or functional integration as indicators of consciousness. The phenomenological stance adopted here does not deny the relevance of such factors, but begins instead from a different ontological commitment: that consciousness is not, in the first instance, a system output or informational relation, but a lived structure, one that is pre-reflectively given and embodied in experience. While accessibility and reportability may be epistemically significant, they are secondary to the lived, situated givenness that constitutes what it is like to be conscious.

Theories such as IIT and PCT mark a partial departure from purely classical representationalist models and, in some respects, show promising alignment with phenomenological concerns – particularly in their emphasis on subjective experience and the dynamic interaction between agent and environment. Yet despite their theoretical ambition, they too often under-specify the role of pre-reflective self-acquaintance and contextual intentionality. In particular, IIT appears to overlook the role of attentional agency in its account of consciousness. Thus, while the critiques that follow focus more directly on GWT and HOT as paradigmatic of access-based models, the explanatory limitations of IIT and PCT are also addressed, especially insofar as none of these frameworks, on their own, fully captures the richness of conscious experience as it is revealed in phenomenological analysis.

### 4.1 Global workspace theory

Global Workspace Theory (GWT), first proposed by Baars (1988, 1997), models consciousness as the result of global broadcasting within a cognitive architecture. According to this theory, numerous specialized processors in the brain operate unconsciously in parallel, but when particular information becomes sufficiently salient, it is amplified and broadcast across a central “workspace,” enabling conscious access. Consciousness, on this view, is not tied to any specific brain region but arises from the large-scale availability of information across distributed systems.

With the advent of neuroimaging, GWT evolved into the Global Neuronal Workspace Theory (GNWT), offering a biologically grounded version of the model. GNWT posits that conscious access emerges through recurrent interactions among high-level cortical regions – particularly the prefrontal, parietal, and cingulate areas. When neural representations reach a threshold of activation, they initiate a “global ignition” event that correlates with conscious awareness. Empirical studies using EEG and fMRI have supported this framework, showing that conscious perception is marked by large-scale, coordinated activation across frontal-parietal networks.

#### 4.1.1 Critiques of global workspace theory

Let us take a deeper look at what GWT considers to be ‘consciousness'. From the outset, GWT was developed to account primarily for Access consciousness (A-consciousness). In a widely cited distinction, Block (1995) characterizes A-consciousness as the availability of information for reasoning, report, and control of behavior, in contrast to Phenomenal consciousness (P-consciousness), which refers to the qualitative, subjective “what-it-is-like” aspect of experience. GWT adopts this distinction and tends to focus its explanatory efforts on A-consciousness. As Baars (1997) illustrates: “While you are conscious of words in your visual focus, you surely did not consciously label the word ‘focus' just now as a noun …” (Baars, 1997, p. 5). What becomes conscious, in this view, is the information that enters the global broadcasting system and becomes available for cognitive access. Mashour et al. (2020) make this orientation explicit: “Access consciousness refers to the fact that conscious information, unlike unconscious information, is accessible to numerous cognitive processors… Thus, unless otherwise specified, the term ‘consciousness' in this review will be replaced by conscious access” (Mashour et al., 2020, p. 776). Here, the *availability* of information, rather than the *raw feel* of experience, becomes the central explanandum of GWT. Or more precisely, GWT tends to conflate raw experience with access itself. Moreover, according to Dehaene and Naccache (2001, p. 7), attention is considered a prerequisite for conscious access.

Block himself later acknowledged that these two forms may not be entirely separable in practice – that is, P-consciousness can become A-consciousness, and vice versa (Bayne and Montague, 2011, p. 8). However, from a phenomenological standpoint, the issue is not merely whether A-consciousness and P-consciousness can always be neatly distinguished, but whether a theory that privileges accessibility can adequately account for the lived, qualitative dimension of experience. The claim that consciousness depends on cognitive availability stands in tension with phenomenological insights. Pre-reflective self-acquaintance, precisely because it is pre-reflective, does not require access in the sense of reportability, inferential integration, or higher-order monitoring. It is not something we attend to, but something through which we are already situated in experience. In other words, the subject is always tacitly aware of itself in the act of experiencing, without needing to cognitively “access” that awareness as an object. This kind of immediate self-givenness precedes any cognitive availability and is often occluded by attempts to explain consciousness in purely functional or representational terms. This divergence reflects more than a difference in emphasis – it reveals a fundamental philosophical divide with direct implications for the explanatory aims of consciousness research.

It is also important to note that Block's distinction between A-consciousness and P-consciousness has been interpreted by some as compatible with a broadly representationalist framework, in which mental representations of externally existing objects underlie cognitive access. While Block does not explicitly frame P-consciousness as representational, his notion of A-consciousness as information functionally available for reasoning, report, and behavioral guidance lends itself to representationalist interpretations. GWT, in adopting this model of access, inherits its functionalist and information-processing commitments. Phenomenology, by contrast, rejects the premise that consciousness can be explained as access to internal content, and likewise denies the dichotomy between an internal realm of representations and an external world of objects. As discussed in Section 2, it frames consciousness not as access to internal representations, but as a mode of intentional engagement that is at once structurally constituted and pre-reflectively given. From a Husserlian standpoint, consciousness is grounded in a priori structures of intentionality and self-acquaintance, while later phenomenologists have further emphasized its embodied, situated, and relational dimensions.

From a phenomenological perspective, we may now ask whether A-consciousness alone can adequately capture the holistic structure of consciousness. According to GWT, consciousness is essentially a matter of access. But can the personal and attitudinal stance toward objects and others—*subjective stance* toward the world, including the *pre-attentive familiarity* with one's own experience—be fully captured by accessibility alone? The concept of phenomenological intentionality cannot be reduced to A-consciousness. Subjective intentionality necessarily involves not only intentional *matter* but also intentional *quality*. The conscious act of “me perceiving a half-filled glass of water” is shaped not merely by the visual sensation of the object, but by my evaluative stance toward it. Indeed, different intentional qualities result in qualitatively distinct experiences, as though a different object is being presented altogether (Husserl, 2001, Vol. II, p. 119–120).^13^

Can subjective feelings of ‘liking' or ‘disliking'—attitudes shaped by optimism or pessimism—be explained purely in terms of awareness or attention? Can GWT account for how such stances alter the conscious act of perceiving the same glass? A-consciousness, concerned solely with whether a representation is accessible, seems inherently incapable of capturing more than intentional matter. To do so, it would need to reframe intentional quality as yet another form of intentional matter. For example, ‘liking' or ‘disliking' might be explained as becoming aware of one's evaluative stance through meta-discovery – a move that echoes the explanatory strategy found in some versions of HOT. This line of reasoning is problematic, first because it introduces infinite regress, and second because it contradicts the very notion of intentional quality. Intentional quality is not derivative; it is a constitutive condition of lived experience. No matter how reflective one becomes of one's attitudes, these reflections are not fully transparent in the way Access consciousness assumes. They are always already shaped and distorted by subjectivity. Conscious subjects are, from the beginning, embedded in and inseparable from their own perspectival stance.

Furthermore, GWT seems to concentrate on cases involving minimal expressions of subjectivity. Much of the literature relies on sensory stimuli perceived through sensory modalities as paradigmatic examples of experience, effectively treating *sensory stimuli under awareness* as its main explanandum. While this offers methodological convenience for empirical research relying on reportable data, it risks simplifying the deeper structures of intentionality. Consciousness must also be explained in cases where subjectivity is not merely present but indispensable – that is, in experiences that could not even arise without subjective participation. As discussed in Sections 2 and 3, sensory stimuli do not constitute the foundational elements of perception or consciousness; rather, they are theoretical abstractions. If GWT fails to incorporate the complex dynamics between embodied subjects and their environments (i.e. dynamics foundational to subjectivity), it risks becoming less a theory of consciousness and more a model of attentional stimulus processing.

GWT's explanatory framework, then, appears insufficient for capturing the full scope of consciousness. It defines consciousness narrowly, as that which is accessible to awareness. While it may account for contextual and spatiotemporal properties of conscious experience through global neuronal broadcasting, it remains confined to representations of intentional matter. By identifying A-consciousness with consciousness *per se*, GWT limits its theoretical reach to neurobehavioral correlates. In this sense, what it explains is closer to “information availability during wakefulness” than to “the emergence of subjectivity as lived experience.” This may seem to address the question of how conscious experience arises, but in truth, it reduces the problem to a narrow functional subset, thereby overlooking the deeper issue of subjectivity. To account for the phenomenological variations of lived experience, particularly those involving intentional quality, GWT must broaden its theoretical scope beyond access-based modeling. After all, GWT is not a representationalist philosophy *per se*, but a functional framework that draws upon representationalist assumptions to interpret neuroscientific data.

Despite these considerations, GWT appears to maintain a strong commitment to representationalist assumptions, thereby sidelining phenomenological insights. In phenomenology, concepts such as pre-reflective self-acquaintance and intentional quality are not equivalent to attention and accessibility, but are fundamentally prior. Accepting the latter as explanatorily sufficient effectively forecloses the former and returns us to the specter of physical reductionism. Even if GWT argues that qualitative, subjective experience can be fully explained in functional terms—that is, that P-consciousness is reducible to A-consciousness—this does not constitute a genuine solution to the problem of consciousness. Even if we eliminate qualia and relocate phenomenality from the “internal mind” to the “external brain,” the explanatory gap remains. As Rudd (1998) notes, the Hard Problem does not arise merely because experience is difficult to explain physically; it arises because subjective experience is undeniably real. Declaring it to be an illusion or functionally redundant does not erase its presence. It remains experientially given, here and now. Any theory that overlooks this essential feature of subjectivity cannot offer a complete account of consciousness.

### 4.2 Higher-order theory

Higher-Order Theories of Consciousness (HOT) posit that a mental state is phenomenally conscious only if the subject is aware of being in that state. This idea is formalized in the *Transitivity Principle*, which holds that a mental state becomes conscious only when it is the object of a higher-order representation. In this framework, consciousness is not intrinsic to first-order perceptual states; rather, it emerges when such states are meta-represented at a higher cognitive level. As such, unconscious perceptual processing is not only possible but widely acknowledged within HOT, with classic examples including absent-minded driving and blindsight (Armstrong, 2002; Carruthers, 1989; Peters et al., 2017).

The HOT family encompasses a range of models unified by their endorsement of the Transitivity Principle, but they differ in how the higher-order representation is construed. Some accounts propose that these are thoughts (Higher-Order Thought theories), while others treat them as perceptual in nature (Higher-Order Perception theories), with recent models exploring hybrid forms (Rosenthal, 1997; Brown et al., 2019; Lau and Rosenthal, 2011). All versions involve at least two levels of representation: a first-order perceptual state and a second-order meta-representation that confers conscious status. Empirical support for HOT often draws on cases of visual awareness (e.g. binocular rivalry, blindsight), but the theory has also been extended to affective and emotional states (LeDoux and Brown, 2017). For a more detailed comparison of multiple HOT models, see Table 1 in Lau and Rosenthal (2011) and Table 1 in Brown et al. (2019). In the discussion that follows, I treat HOT as a general class of theories without committing to any specific subtype.

#### 4.2.1 Critiques of higher-order theory

Higher-Order Theories of consciousness have long stood in tension with phenomenology, particularly in their treatment of self-consciousness. In HOT, object-directed consciousness is categorized as *transitive consciousness*, while pre-reflective self-consciousness is viewed as *intransitive consciousness*. Crucially, HOT posits that intransitive consciousness is not intrinsic but derivative – it arises only through the existence of transitive consciousness. That is, self-consciousness is taken to emerge from consciousness that is directed at or reflective about an object, making it thoroughly dependent on the existence of *object-consciousness* (Rosenthal, 1986). This stance, similar to GWT's presupposition of A-consciousness as the necessary precondition for all conscious states, stands in clear contrast to phenomenological accounts.^14^

Consequently, many of the phenomenological critiques raised against GWT apply equally to HOT. A phenomenologist would likely invert the explanatory order, proposing that intransitive consciousness is the precondition for transitive consciousness. This view is strongly supported by Zahavi, who argues that pre-reflective self-consciousness is not a byproduct of higher-order monitoring but an intrinsic feature of experience itself. On his account, all conscious states are tacitly self-aware in a non-objectifying way, and any theory that fails to account for this immediacy risks misrepresenting the structure of subjectivity. Rather than treating self-consciousness as a representational add-on, Zahavi (2005, p. 17–29) emphasizes its foundational role in enabling experiential coherence and first-personal perspective. On this view, the foundation of conscious experience lies not in the Transitivity Principle, but in what might be called the Intransitivity Principle (this phrase is used here heuristically). For phenomenology, the composition of conscious life does not begin with object-directed content but with the lived structure of subjectivity itself. Metaphorically, subjectivity functions not as the spotlight that illuminates content, but as the stage that makes any performance possible. In other words, for there to be a spotlight in the mental theater, the theater must already exist.^15^

Moreover, as the term “pre-reflective” self-consciousness itself indicates, phenomenologists do not regard self-acquaintance or self-intimacy as a higher-order mental activity that observes or monitors one's own mental states. In Husserl's formulation, it is non-observational and directly *lived through (Erlebt)*; it is neither internal perception nor reflective thought (Husserl, 2001, Vol. II, 6th Investigation). The term *lived experience (Erlebnis)* here refers not merely to the fact that experience is first-personal, but to the way it is directly undergone prior to reflection, observation, or conceptualization. To experience an object in this sense means undergoing it at the same hierarchical level, rather than recognizing it from a detached, higher-order cognitive position.^16^ From a phenomenological perspective, pre-reflective self-consciousness, or “intransitive” consciousness, is posited as an irreducible and intrinsic feature of all conscious states. Critics from the HOT tradition have argued that treating such properties as irreducible renders them beyond scientific or theoretical inquiry, effectively mystifying consciousness (Rosenthal, 1993). But this critique invites a parallel concern: by positing the Transitivity Principle as a foundational feature of consciousness, does not HOT also posit a limit beyond which no further reduction is possible? At its core, the Transitivity Principle treats “orientation toward an object” as intrinsic to conscious experience – a move not fundamentally different from phenomenology's claim about subjectivity.

While both HOT and phenomenology acknowledge that consciousness has essential features, they fundamentally disagree on what those features are and how they should be explained. What may appear as a perspectival difference ultimately reflects contrasting commitments to the structure of subjectivity: whether self-consciousness is intrinsic and immediate, or whether it emerges through representational reflection. From the HOT perspective, the phenomenological emphasis on irreducible subjectivity is often seen as obscuring the conditions for scientific or theoretical analysis. However, recognizing something as irreducible does not mean giving up on explanation. In fact, phenomenology takes the irreducibility of pre-reflective self-consciousness as the starting point for a rich explanatory project. As outlined in Sections 2 & 3, it can account for how consciousness becomes contextually structured from self-intimacy to spatial, temporal, intersubjective, and even object-directed experiences such as attention, which HOT emphasizes. If one framework offers a broader and deeper explanatory reach, there may be good reasons to treat it as a more promising explanatory framework.

### 4.3 Integrated information theory

Integrated Information Theory (IIT) offers a computational framework for explaining consciousness by treating qualia as intrinsic informational properties of physical systems. Instead of focusing on neural correlates of consciousness (NCC) or observable behavior, IIT starts with the assumption that consciousness is ontologically primary and aims to characterize it in formal, quantitative terms. The theory begins with a set of *phenomenal axioms*—Existence, Intrinsicality, Information, Integration, Exclusion, and Composition—which are intended to reflect universally agreed-upon features of subjective experience (Albantakis et al., 2023).^17^ These axioms are then mapped onto *physical postulates*, which specify the structural and causal requirements a physical system must satisfy in order to instantiate those phenomenal features. IIT's ultimate goal is to compute a measure of consciousness, denoted as Φ, that quantifies both the presence and qualitative character of experience within a given physical substrate (Tononi, 2005; Tononi and Koch, 2015; Albantakis et al., 2023).

Central to this computation are the notions of *intrinsic information* and *integration*. Intrinsic information is defined as the informational difference between the causal (constrained) and non-causal (unconstrained) states of a system, distinguishing it from Shannon information. Integration refers to the irreducibility of the system's informational structure: a system is said to be integrated if it generates more information as a whole than the sum of its parts. The amount of integrated information lost upon partitioning the system determines the value of Φ, which, according to the theory's identity assumption, is identical to consciousness itself. In its most recent formulation, IIT introduces the concept of a maximally irreducible conceptual structure (Φ_*max*_), whose geometric and quantitative features are taken to correspond directly to the quality of experience (Moon and Pae, 2019; Albantakis et al., 2023).

#### 4.3.1 Critiques of the integrated information theory

Integrated Information Theory (IIT), though grounded in neuroscience, takes an unusual and ambitious turn by placing P-consciousness rather than A-consciousness at the center of its explanatory framework. It aims to preserve the intrinsic, ineffable, and private character of qualia, engaging directly with the so-called Hard Problem of consciousness. Unlike many scientific theories that either sideline or reinterpret P-consciousness in terms of functional accessibility or cognitive report, IIT treats it as the entirety of consciousness. This orientation is reflected in its foundational claim: “That experience exists—that ‘there is something it is like to be'—is immediate and irrefutable, as everybody can confirm, say, upon awakening from dreamless sleep” (Albantakis et al., 2023, p. 3). As commendable as this emphasis may be, it raises a critical concern: how much explanatory traction can be gained by building an entire theory of consciousness around P-consciousness alone, while marginalizing other dimensions of experience?

Even if P-consciousness are central to understanding consciousness, they do not exhaust its full structure. Just as GWT risks reducing consciousness to cognitive accessibility, IIT may err in the opposite direction by identifying it wholly with qualia, understood as intrinsic, privately accessible phenomenal properties. While phenomenology emphasizes first-person experience, it does not equate it with raw sensation; living-through *(Erlebt)* involves structures that extend beyond mere qualitative feel. The issue is not that IIT fails to naturalize phenomenology as it does not aim to be a phenomenological theory, but that its conception of consciousness is overly narrow, even on its own terms. (Indeed, IIT researchers explicitly acknowledge that their phenomenal axioms are not derived from classical phenomenology – see text footnote ^17^.) As a result, IIT's focus on qualia/P-consciousness risks collapsing the complexity of conscious process into a certain internal informational configuration, leaving out dimensions such as embodiment, intentionality, and intersubjective constitution that phenomenology regards as essential.

IIT constructs a formally rigorous model in which qualia are understood as informational entities, grounded in five phenomenal axioms: Intrinsicality, Information, Integration, Exclusion, and Composition.^18^ These axioms are translated into physical postulates that define the structural properties a system must possess in order to generate consciousness, understood as integrated information, Φ. Since Φ is computed from the intrinsic causal structure of the system that is independent of external input, it is framed as an inherent and self-contained property. In this way, IIT aims to capture the ineffable and private nature of qualia through formal means. The problem, however, is that IIT treats its account of qualia as sufficient for explaining consciousness as a whole. As a result, its model tends toward a solipsistic conception: because Φ is necessarily intrinsic, the conscious system is portrayed as fundamentally self-contained, capable of being conscious independently of any relation to the world. This stands in marked contrast to the phenomenological account, as discussed in Section 2. From that perspective, subjectivity is not a sealed inner realm but something constituted dynamically through situational context, embodied interaction, and recognition of others as fellow agents. It is not enough for consciousness to exist in itself; it must also exist *in the world*. In this sense, subjectivity cannot be fully established without the presence of others and a shared environment. The relational structure of lived experience—its intentional orientation, temporal openness, and intersubjective constitution—is absent from IIT's conception of consciousness, which remains enclosed within the causal borders of the system it describes.

Still, there is conceptual space within IIT that could open it to a richer account of subjectivity. Phenomenology acknowledges that selfhood is not reducible to social or linguistic constructions alone. While early phenomenologists emphasized its intrinsic, pre-reflective character, later developments have increasingly recognized the formative role of embodiment, intersubjectivity, and cultural-historical context in shaping subjective experience. Across these variations, a consistent theme remains: the emphasis on pre-reflective self-acquaintance as a minimal yet irreducible feature of conscious mental phenomena. IIT, for its part, posits *Existence* as the most fundamental of its axioms—described as “The One Axiom” or “the ultimate zeroth axiom”—which underlies all subsequent phenomenal axioms (Albantakis et al., 2023, p. 3). This formulation may suggest a structural parallel with the phenomenological notion of first-person givenness.

However, upon closer inspection, IIT's axiom of Existence lacks reference to self-acquaintance or first-personal intentionality. It states that “experience exists” but does so in abstract and impersonal terms, omitting any sense of *mineness* or self-involvement. In attempting to explain the structure of qualia, IIT does not engage the core condition that makes experience subjective in the first place. From a phenomenological perspective, any account of existence that bypasses this minimal form of self-consciousness falls short of describing what it means to live an experience. The intimacy of lived experience *(Erlebnis)* cannot be captured by a merely structural or computational account of informational properties, as it arises through a continuous and dynamic interaction with the world – an unfolding process shaped by embodiment, temporality, and intersubjective engagement. Thus, although IIT claims to reflect phenomenological intuitions, its axioms ultimately interpret the features of consciousness as outputs of causal and informational processes, rather than as expressions of lived, intentional structure.

Consequently, IIT not only struggles to account for the subjectivity of qualia in a non-solipsistic manner, but also fails to capture the broader range of conscious processes in which qualia are embedded. While IIT aims to capture the structural complexity of conscious experience through concepts like integration and composition, its formalized approach risks neglecting the dynamically situated and context-sensitive dimensions of experience. These include the dynamic interplay between central and peripheral awareness, the temporal and evaluative structures of intentionality (such as anticipation, recollection, and valuation), and the reflexive self-awareness of the conscious agent. Any comprehensive theory of consciousness must address these elements.

IIT's treatment of qualia as informational correlates is, without doubt, an ambitious and creative proposal. But qualia alone do not constitute the whole of conscious experience. Without integrating the notion of a self-aware agent engaged with the world, IIT's model risks fragmenting consciousness into decontextualized informational structures that are far removed from lived experience. Given that many facets of consciousness can be meaningfully explained through intentionality, it becomes clear that qualitative and functional aspects are not mutually exclusive but co-constitutive. Indeed, while IIT's phenomenal axiom seeks to preserve the immediacy of experience by resisting reduction to function, it ironically risks producing the very fragmentation it aims to avoid. Its axioms, though innovative, may ultimately function not only as guiding principles but also as limiting constraints on the theory's explanatory reach.

### 4.4 Predictive coding theory

Predictive Coding Theory (PCT), also known as Predictive Processing Theory or Active Inference framework, understands consciousness as emerging from inferential processes grounded in active engagement with the environment. Drawing on Helmholtz's notion of unconscious inference, PCT suggests that the brain operates as a hierarchical prediction machine, constantly generating and updating internal models to minimize the mismatch between sensory inputs and prior expectations (Seth, 2015; Metzinger and Wiese, 2017). Through its integration with the Free Energy Principle (FEP), PCT formalizes this idea by proposing that biological systems, including the brain, minimize free energy to resist entropy and maintain functional coherence (Friston, 2010, 2012). Perception and action are thus conceptualized as complementary strategies for minimizing prediction error across multiple levels of processing, with the brain functioning as a hypothesis-testing system (Hohwy, 2012). This framework is often illustrated through the “control room” analogy, in which an agent must infer the state of the world based solely on internal readouts – a metaphor for the brain's reliance on internal generative models rather than direct access to reality (Metzinger and Wiese, 2017).

On this view, consciousness is not a passive imprint of sensory data, but an actively generated simulation shaped by probabilistic inference. The generative model maintained by the brain encodes hypotheses about hidden causes in the world and is continuously refined through the dynamics of prediction error minimization (Friston, 2013b, 2018). Conscious awareness is thus understood in terms of how ascending prediction errors revise high-level priors, thereby shaping the phenomenal character of experience. Although PCT does not consistently refer to “state consciousness” in explicit terms, certain interpretations—particularly those aligned with the Free Energy Principle—suggest that consciousness may arise when prediction errors become globally accessible and integrated into deeper representational systems.^19^

#### 4.4.1 Critiques of the predictive coding theory

One of the most notable strengths of PCT lies in its emphasis on environmental interaction as central to conscious processing, offering a promising framework for modeling consciousness as the activity of an embodied agent embedded in the world. However, many traditional formulations of PCT retain a Helmholtzian perspective on inference, which presupposes a sharp division between internal cognitive states and an external, perceivable world. This dualistic paradigm, which dichotomizes the subject (animal) and its environment, tends to limit the theory's explanatory reach, as it proceeds independently of the intrinsic properties of subjectivity. While PCT can account for types of consciousness that are representationally accessible, it struggles to address non-representational aspects such as pre-reflective self-acquaintance. This omission renders such dimensions of experience theoretically irrelevant or invisible within the standard inferential framework.

Encouragingly, not all interpretations of PCT are committed to this Helmholtzian model. Some theorists reinterpret active inference not as the construction of an inner world-model *per se*, but as a generative and world-involving process in its own right. As Seth notes: “Linking these origins to their modern expression in Karl Friston's ‘free energy principle' (2010), perception emerges as a consequence of a more fundamental imperative toward homeostasis and control, and not as a process designed to furnish a detailed inner ‘world model' suitable for cognition and action planning.” (Seth, 2015, p. 3). This opens up the possibility of reconceiving perception as a constitutively relational rather than representational process, which is a shift that aligns PCT more closely with the spirit of embodied and enactive phenomenology, as emphasized in the work of Varela et al. (1991), Thompson (2007), Gallagher (2005), and Clark (2013).

PCT characterizes conscious perception as arising from the brain's ongoing inference of its situated context in the world – an account that appears well-suited to explain the world-involving dimensions of intentionality emphasized in phenomenology. However, it is important to note that such ongoing inference of context does not straightforwardly or self-evidently lead to the emergence of consciousness. PCT lacks a clear justification for why active inference should necessarily give rise to conscious experience. While PCT's framing of perception as an embodied and self-organizing process may offer a structural foundation for minimal selfhood or situated subjectivity, it does not in itself explain why such interactions should be experienced as subjectively lived. In other words, the mere modeling of one's own body and surrounding environment is not obviously sufficient to account for the pre-reflective givenness of experience. This limitation ultimately reflects a deeper challenge for PCT: the absence of a principled account of why generative inference should give rise to consciousness at all.

While some versions of the theory treat mental states as emergent properties of predictive processes (Seth, 2015; Friston, 2016), it remains unclear how and why these emergent properties must entail subjectivity. For instance, GWT grounds its claims in empirical findings on global neural broadcasting, and IIT articulates mathematically defined conditions under which qualia are said to arise. PCT, however, offers a compelling picture of inference dynamics but does not demonstrate how such dynamics suffice to bridge the explanatory gap between unconscious computation and conscious experience. The Free Energy Principle may show how inference occurs spontaneously in biological systems, but spontaneity alone does not resolve the philosophical burden of showing why such processes result in consciousness rather than merely functioning behavior. Unless PCT can provide a principled account of why inference should lead to subjective experience, it risks being classified not as a theory of consciousness but as a biologically inspired model of adaptive regulation.

That said, if a defensible principle of emergence could be articulated, PCT might offer strong support for phenomenological insights by providing a scientifically grounded account of consciousness. Its inferential mechanisms could plausibly align with the forms of self-acquaintance and contextual embodiment discussed in Section 3. Indeed, PCT has already been applied to areas ranging from temporality and attention to the modeling of agent-based cognition (Sandved-Smith et al., 2021; Ramstead et al., 2022; Albarracin et al., 2023; Bogotá and Djebbara, 2023). However, without systematic theoretical postulation and empirical validation, such efforts risk remaining speculative or merely analogical. If PCT is to serve as a rigorous theory of consciousness, it must do more than model functionally adequate behavior: it must explain why and how inferential processes give rise to lived experience.

## 5 Reconciling empirical theories of consciousness

**Figure 1:**
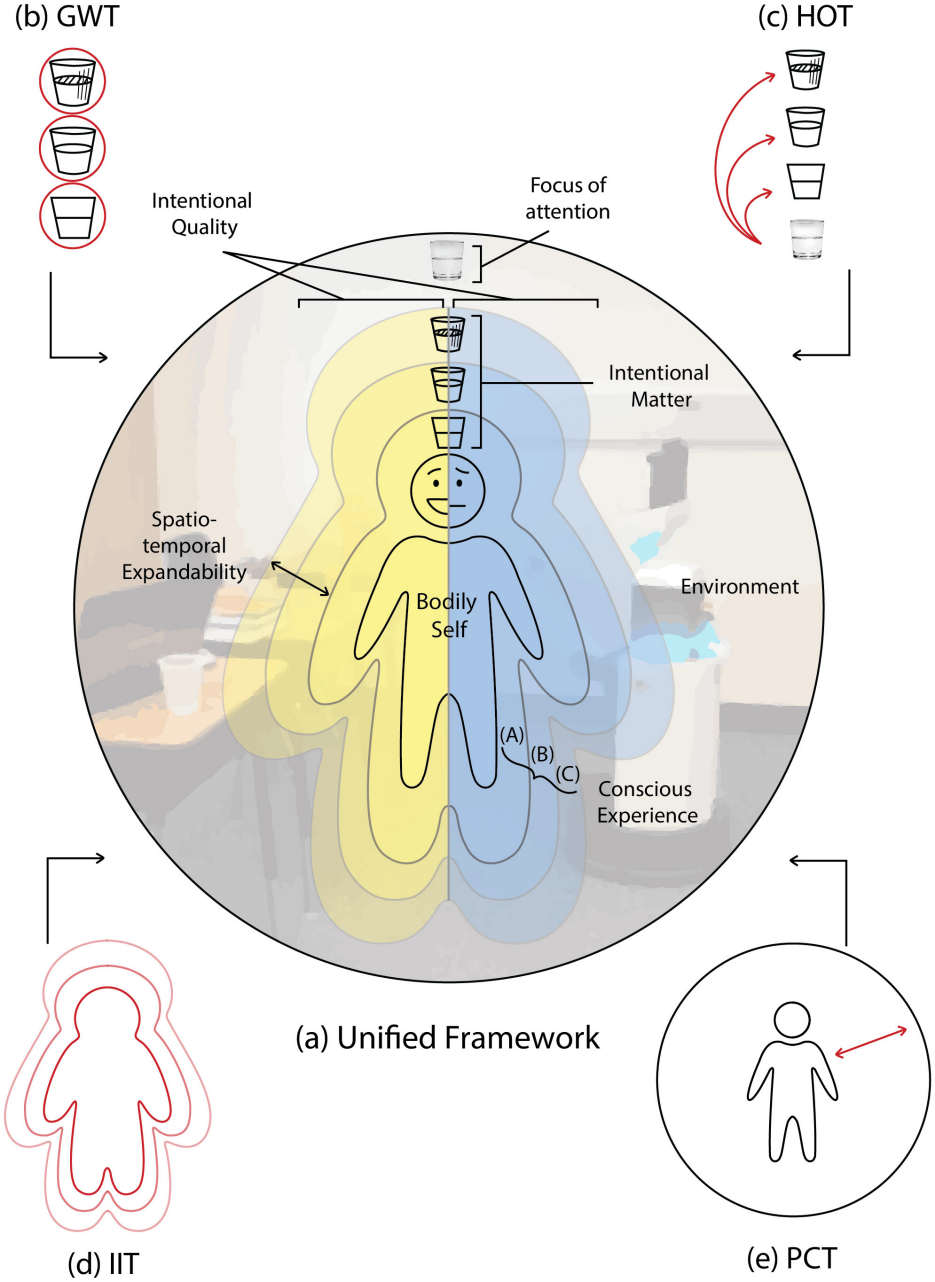
Unified framework for consciousness theories. **(a)** Unified framework. This diagram integrates major phenomenological insights into a unified structure. It depicts a conscious agent situated in a controlled environment, attentively focusing on a half-filled glass of water. The agent perceives this object from two contrasting attitudinal perspectives—pessimistic (shaded blue) and optimistic (shaded yellow)—highlighting variations in intentional quality. Although directed at the same intentional matter, these perspectives yield distinct conscious experiences. The depth and vividness of experience vary with the agent's degree of engagement, represented as a series of layered contour surfaces radiating from the bodily self at the center. This structure reflects the gradation from pre-reflective self-acquaintance at the core to fully articulated perception. For instance, peripheral awareness results in a less vivid experience (A), while active engagement intensifies phenomenality (C), illustrating the dynamic constitution of conscious experience. **(b)** Global workspace theory (GWT). GWT is mapped onto this framework as the mechanism by which attentionally selected content becomes globally accessible. It aligns with the representation of Access consciousness, wherein the content of focus is broadcast across cognitive systems, enabling coordinated behavioral and cognitive responses. **(c)** Higher-order theory (HOT). HOT is mapped here as a hierarchical layer over intentional content. According to the transitivity principle, a mental state becomes conscious only when it is represented by a higher-order state. This depiction reflects the core claim of HOT, in which meta-representational processes are taken to determine conscious status within the model's representational architecture. **(d)** Integrated information theory (IIT). IIT is represented at the level of structural integration. Rather than emphasizing specific intentional contents, it accounts for the overall compositional structure of experience. Within the framework, IIT corresponds to the topographical “surface” of the contour layers, expressing the intrinsic integration of phenomenological elements within the system. **(e)** Predictive coding theory (PCT). PCT is represented through the dynamic interface between the bodily self and its environment. It highlights how ongoing agent-environment interactions, mediated by active inference and predictive engagement, shape and modulate the evolving structure of conscious experience.

Up to this point, I have examined how four major scientific theories—GWT, HOT, IIT, and PCT—define consciousness and approach its explanation. I also considered how these approaches differ from the structures of experience revealed through phenomenological reflection. Figure 1a outlines the central features of consciousness discussed in Section 3. Figures 1b–e provide a schematic comparison of how each theory captures certain dimensions of this structure. GWT emphasizes representational access, grounding its account in neural mechanisms of broadcast. HOT focuses on meta-representational access as the condition for consciousness. IIT approaches the issue from the standpoint of intrinsic informational structure, while PCT frames consciousness as a byproduct of biologically embedded inference processes.

However, as the critical discussions above suggest, no single theory offers a comprehensive account of consciousness in its full complexity. Even the features outlined in Section 3, while central, are not proposed as exhaustive. From this perspective, the minimal conditions for consciousness offered by each theory reflect only partial facets of a larger phenomenon. In this light, the prevailing tendency to stage these theories in direct opposition, debating which one “gets it right,” may itself be misguided. Such theoretical rivalries risk obscuring the deeper conceptual work needed to clarify what each theory is actually trying to explain.

What we need, I suggest, is a shift in orientation: from theoretical rivalry to conceptual integration. I believe that a meaningful integration of these theories is both possible and desirable, without necessarily giving rise to conflict. At the same time, such a fusion should not be mistaken for a comprehensive solution to the problem of consciousness. Rather, it may serve as a constructive starting point – a way to frame the diversity of conscious phenomena without prematurely foreclosing theoretical possibilities. Each theory brings attention to a distinct aspect of consciousness, shaped by its explanatory commitments. If consciousness involves both subjective self-acquaintance and access-based awareness, situated within embodied and environmental contexts, then it becomes possible to see these approaches as complementary rather than mutually exclusive. This is the perspective illustrated in Figure 1a. Rather than choosing one model over another, we might consider how each contributes to a broader and more flexible understanding of conscious phenomena.

To reiterate, these theories need not compete; they can coexist in parallel, as each illuminates different dimensions of consciousness. What is needed is a movement toward a more comprehensive and integrative approach. One promising path is to respect the explanatory boundaries of each theory while adapting their methods to account for distinct subdomains of conscious phenomena. By doing so, we may begin to stitch together a more cohesive framework that draws from the strengths of existing models. In the remainder of this section, I examine how each theory corresponds to specific phenomenological features, and I suggest possible conceptual adjustments that could allow for their integration without abandoning phenomenological insight. Finally, I introduce a selection of studies that reflect a similar integrative outlook, highlighting concrete efforts toward bridging disparate theories of consciousness.

### 5.1 Reducible properties of consciousness

GWT and HOT share a broadly reductionist orientation that aligns with representationalism. Both theories emphasize the role of representational awareness in the emergence of consciousness and define conscious states in terms of functional access. This alignment stems from a shared assumption that conscious states can be fully explained by reference to internal representations and their functional roles within a system, thereby reducing phenomenality to representational accessibility. This contrasts with phenomenological methodology, which does not typically begin by separating the subject from the world. Instead, it treats phenomenal experience, including its intentional and embodied dimensions, as the primary object of inquiry. In this section, however, I explore how GWT and HOT might coexist with phenomenology and other models, provided we understand their explananda as addressing specific subsets of the broader phenomenon of consciousness.

GWT proposes that consciousness arises through a process of global broadcasting, whereby information becomes accessible across cognitive systems. In this view, the critical unit is not neural activation itself, but the integrated mental structure represented by such activity (Baars, 1997). When we turn to GNWT, however, the emphasis shifts toward the neurobiological mechanisms that instantiate this process. Global broadcasting here refers to specific neural ensembles whose activation marks the transition into conscious awareness (Dehaene et al., 2006). These two perspectives are not contradictory; rather, they reflect different explanatory levels. GWT, in its broader sense, captures the cognitive structure of conscious content, while GNWT focuses on the underlying neural dynamics. Taken together, they show how A-consciousness can be studied at both the functional and biological levels.

Because GWT focuses primarily on those aspects of consciousness that are functionally explicable, it offers a relatively accessible and empirically tractable framework. Its explanatory strength lies in identifying the neural conditions necessary for A-consciousness to arise. As illustrated in Figure 1b, GWT explains how intentional matter becomes globally available to cognitive systems through mechanisms of neural broadcasting, regardless of the specific qualitative content of the target. In this respect, GWT offers a broad explanatory scope for a wide range of conscious phenomena that are behaviorally or reportably accessible.

However, it is important to note that not all instances of global broadcasting necessarily result in reportable conscious experience. Conversely, there are also cases where subjects report a conscious experience even in the absence of globally distributed neural activity (Brown et al., 2019; Rosenthal, 2008). This suggests that GWT's framework may benefit from supplementation. For example, Van Gulick's Higher-Order Global State (HOGS) theory (Van Gulick, 2004) adds an additional condition: that a mental state must be integrated into a higher-order system of self-awareness to qualify as conscious. Such refinements could help GWT better accommodate phenomenal features that it does not, by itself, explain.

Indeed, the neural structures implicated in GWT's account of consciousness (Del Cul et al., 2007; Odegaard et al., 2017) differ significantly from those proposed by IIT (Boly et al., 2017; Koch et al., 2016). This divergence has led to ongoing adversarial collaboration projects aimed at directly comparing the predictive power of the two models (Cogitate Consortium et al., 2023). While this is often framed as a competitive empirical test, it may also be interpreted as a productive comparison of theories with fundamentally different explananda. GWT focuses on access mechanisms and cognitive integration, whereas IIT targets the structural features of phenomenality itself. Rather than seeing this contrast as a zero-sum rivalry, such collaborations might serve to demonstrate how multiple theories can coexist by clarifying the distinct dimensions of consciousness they address.

Just as GWT emphasizes the emergence of an accessible “spotlight” of consciousness, HOT likewise seeks to explain consciousness in terms of accessibility – specifically, through higher-order metacognition. According to HOT, a mental state becomes conscious *only* when it is the object of a higher-order mental state that represents it (Rosenthal, 1997). This criterion introduces slightly stricter phenomenological conditions than those proposed by GWT, which treats consciousness as the sustained availability of representational content across distributed neural systems.

In essence, both theories address A-consciousness, but they differ in the specific features they emphasize. GWT focuses on neural broadcasting and functional availability, whereas HOT centers on the meta-representational structure of conscious states. The distinction lies in whether access alone suffices, or whether awareness of access—i.e. a second-order representation—is required for a state to qualify as conscious. These contrasting emphases are illustrated in Figures 1b, c, particularly in how each theory conceptualizes the transition from unconscious to conscious processing.

Despite addressing overlapping explananda, GWT and HOT are not inherently incompatible. As noted by Brown et al. (2019), the broad accessibility of information does not conflict with the presence of self-referential mental processes. Dehaene et al. (2017) similarly argue that global broadcasting and higher-order metacognitive reference constitute two orthogonal dimensions of conscious processing, both of which are typically active in everyday experience. Since GWT does not specify the mental processes by which the “spotlight of attention” becomes phenomenally salient, it leaves room for compatibility with HOT's account of higher-order awareness.^20^ While GWT places greater emphasis on the neural mechanisms of conscious access and HOT on metacognitive representation, both theories can be viewed as capturing complementary features of Access consciousness. Rather than presenting mutually exclusive accounts, they may together illuminate different layers of how accessible experience is generated and maintained.

One dimension of A-consciousness that is phenomenologically significant is the subject's capacity for volitional and selective attention – the ability to modulate awareness in a goal-directed manner. From a phenomenological perspective, this attentional agency does not require reflective observation of one's mental states; rather, it is lived through directly and pre-reflectively, as part of the ongoing structure of experience. HOT, despite operating within a representational and metacognitive framework, offers a valuable explanatory model for how such agentive modulation might be implemented in cognitive architecture. Although approached from a different angle, HOT's central insight—that certain forms of conscious mental process arise when one becomes aware of one's own mental states—can still be seen as engaging with the same structural phenomenon of attentional selectivity. While the Transitivity Principle remains philosophical disagreement, setting that aside reveals a productive point of contact: both HOT and phenomenology, in their own ways, recognize that consciousness involves the capacity to guide or modulate attention through the subject's own activity. In this sense, HOT's emphasis on higher-order cognition may be read not as a rival to phenomenology, but as a model that highlights certain mechanisms consonant with the agentive structure of conscious experience.

### 5.2 Irreducible properties of consciousness

IIT seeks to reconstruct the question of what it means to be a conscious being by starting not from behavioral or representational correlates, but from the inescapable elements of qualitative experience itself (Albantakis et al., 2023). It is grounded in the conviction that consciousness cannot be fully explained in terms of neural correlates or functional operations alone, especially insofar as these rely on representational assumptions. IIT begins from the assertion that consciousness is not reducible—particularly at the neurobiological level—and that any explanatory framework must preserve the intrinsic character of experience rather than translating it into purely functional or representational terms. In doing so, it focuses on intrinsic features of experience that resist decomposition into simpler or more functionally definable parts. This orientation sets IIT apart from more reductionist approaches, such as GWT and HOT, which begin from functional, accessible, or metacognitive dimensions. If one were to frame these approaches in a zero-sum logic by asking which theory alone could account for consciousness in its entirety, IIT's position would seem difficult to reconcile with its rivals. But such framing is neither necessary nor productive. If we instead view these theories as complementary, each offering insights into different aspects of consciousness, then IIT's distinct focus becomes a valuable contribution rather than a competing claim.

Whereas GWT and HOT focus on how information becomes globally available or metacognitively accessible, IIT turns its focus to the phenomenal textures themselves. As illustrated in Figure 1d, IIT may be seen as addressing the surface layers of conscious experience, layered around the bodily self like a Russian doll. Its concern is not with how experiences are functionally produced or accessed, but with what intrinsic properties they must have in order to be what they are, to appear as they do. Starting from the brute fact of experience (“there is something it is like”), IIT aims to identify the informational structures that such experiences entail (Tononi et al., 2016). From this foundation, it attempts to work backward toward the physical or neurobiological configurations that could instantiate such informational properties. This approach resonates with phenomenological methodology, which also begins not with representational function, but with the lived givenness of experience as the proper object of investigation.

While the axioms at the heart of IIT are not phenomenologically grounded in a strict philosophical sense (see Bayne, 2018 for detailed critiques of each axiom), the overall approach of the theory nonetheless shows a methodological affinity with phenomenology, insofar as it takes the structure of subjective experience as its starting point. IIT emphasizes features such as the intrinsic, unified, and richly differentiated character of experience, which resonate with certain structural descriptions found in reflective analysis. That said, IIT's model remains primarily oriented toward the study of sensory experience and does not explicitly engage with dimensions such as temporality or pre-reflective/reflective self-awareness. Nevertheless, it offers a compelling framework for modeling certain aspects of P-consciousness that remain underdeveloped in many other theories. Rather than attempting to expand IIT's scope prematurely, one promising path may lie in modestly supplementing it. For example, a limited principle borrowed from HOT—such as the idea that certain conscious contents become phenomenally salient through higher-order access—could be used to account for those situations in which P-consciousness and A-consciousness coincide. This would not replace IIT's core assumptions, but offer an auxiliary condition relevant in cases such as lucid awareness, without assuming that all phenomenality is dependent on access. Such integrative steps could help clarify the relations between different theories while preserving the methodological integrity of each.

### 5.3 Embodied properties of consciousness

PCT, in particular, offers a theoretical structure well-suited to explaining the conscious subject as an embodied agent, especially in interpretations that emphasize perception and action as environmentally embedded processes. Among the theories of consciousness examined so far, this strand of PCT appears especially promising for engaging with phenomenological insights in a scientifically tractable manner. Its account of conscious phenomena as emerging from a fundamental hyperprior—understood broadly as the tacit familiarity with one's own bodily presence—and unfolding hierarchically, like ripples expanding in concentric circles (Friston, 2013a), closely mirrors the phenomenological view in which intentionality is grounded in pre-reflective self-acquaintance (see Figure 1e).

For instance, gist perception, as modeled in hybrid predictive coding frameworks (Tscshantz et al., 2023), offers a clear example of perceptual organization that precedes the emergence of focal attention. From a phenomenological standpoint, attentional focus is not required for intentional matter to arise; rather, experience may be pre-structured by background bodily awareness and context-sensitivity. A related case is the uniformity illusion, which, under phenomenological analysis, highlights the situational and interpretive nature of perceptual appearance. PCT helps account for this by showing that prediction updates are primarily driven by foveal sensory inputs, while peripheral signals contribute minimally to the model revision (Otten et al., 2016). This suggests that detailed visual information at the focus of attention serves as an anchor point for the broader perceptual field, resulting in uniformity illusions based on incomplete prediction error resolution. Furthermore, recent developments in PCT have extended its explanatory reach to more temporally complex structures, including Husserlian time-consciousness and the layering of metacognitive attention, within the framework of active inference (Bogotá and Djebbara, 2023). While such models are still in early stages of development, they point to promising lines of convergence between phenomenological insight and computational modeling. In this light, PCT appears especially well-positioned to contribute to a scientifically tractable account of consciousness – one that remains grounded in the structures of lived experience rather than abstracted from them.

Most notably, PCT's modeling framework is marked by a high degree of versatility, owing to its foundation in hierarchical active inference. This structural adaptability opens the door to various computational applications, including its integration into machine learning architectures. For example, the hybrid predictive coding model developed by Tscshantz et al. (2023) illustrates one way in which PCT principles can inform artificial systems modeled on aspects of natural cognition. Importantly, such implementations need not rely exclusively on neural networks. As shown in computational neurophenomenology research by Ramstead et al. (2022), the same inferential principles can be formalized as a hierarchy of Bayesian processes, underscoring the flexibility of PCT in modeling conscious-like behavior within diverse theoretical and technological contexts.

This structural flexibility also points to a deeper conceptual connection between PCT and Perceptual Reality Monitoring (PRM), a framework that may be interpreted as an extension of HOT. Like PCT, PRM employs hierarchical Bayesian inference to model conscious perception (Dijkstra et al., 2022). A related model, Higher-Order State Space (HOSS) model, shares this hierarchical architecture and offers a probabilistic account of how perceptual states are monitored and evaluated. While HOSS may initially resemble Signal Detection Theory (SDT) in tracking “correct representations” through probabilistic inference, it departs significantly by positing a structured hierarchy of higher-order abstract states, something that is not required by SDT (Fleming, 2020). This contrast mirrors the distinction between GWT and HOT, in which the latter introduces additional levels of metacognitive representation (see text footnote ^20^ for further discussion on the link between GWT and SDT). Given these structural parallels, it is plausible that elements such as volitional selective attention could be incorporated into predictive processing frameworks, further enriching the interface between PCT and higher-order theories of consciousness.

Interestingly, PCT and IIT offer complementary perspectives on conscious systems. Whereas PCT articulates principles that are realized through the system's ongoing inferential dynamics, IIT defines consciousness in terms of a structural property—integrated information, denoted as Φ—which quantifies the system's causal irreducibility. Despite these differences, the two frameworks are not mutually exclusive. If a system operating under PCT principles is sufficiently causal (i.e. capable of computing the transition probabilities of its own internal states over time), then in principle, its Φ value can also be computed. This opens up the possibility of applying PCT and IIT in tandem, provided that the system exhibits both dynamic inference and stable causal architecture. Indeed, such integration has already been explored in simulation studies. For example, the animat model presented in Lundbak Olesen et al. (2023) simultaneously applies the Free Energy Principle and IIT to a single artificial agent, calculating both surprisal and integrated information within the same framework. While computing Φ remains computationally intensive under current IIT formulations, these hybrid models illustrate how PCT and IIT might be combined to yield a richer explanatory framework – one that bridges dynamic inference and structural integration in the study of consciousness.

### 5.4 Cases of integrative approaches

Let us now turn to research that resonates with the integrative orientation proposed in this paper. A notable example is Adam Safron's Integrated World Modeling Theory (IWMT) of consciousness (Safron, 2020, 2022). IWMT builds on and extends the foundations of existing theories—most notably GWT, PCT, and IIT—to articulate a more comprehensive framework. At its core, IWMT emphasizes autonomy, or agentic causation, as the basis for consciousness, arguing that conscious systems must not only model their environments but also actively regulate their engagement with them. In this view, consciousness emerges as the experience of being a system that generates and updates its world model in a causally, temporally, and spatially coherent manner. These models serve to guide perception and action while minimizing prediction error. IWMT thus conceptualizes consciousness as the subjective perspective of a self-organizing, informational entity – a world model that maintains internal coherence and environmental adaptability. This perspective not only helps bridge PCT and IIT but also addresses a common criticism of predictive processing accounts: namely, the lack of a clearly specified connection between generative models and the phenomenology of subjective experience.

To realize these ideas computationally, IWMT introduces self-organizing harmonic modes (SOHMs), dynamical structures that emerge from the synchronization of neural activity. SOHMs fulfill core functional requirements of PCT by implementing hierarchical predictive inference, while also aligning with the central tenets of IIT by enabling integration across the system's causal structure. At the same time, SOHMs support key features of G(N)WT by amplifying and broadcasting information across distributed neural assemblies, enabling large-scale coordination. Functionally, they operate as attractor states or eigenmodes that facilitate resonance, signal enhancement, and global access – serving as plausible substrates for phase transitions or ignition events that mark transitions into conscious awareness. In this way, IWMT offers a cohesive account that links the architectural mechanisms emphasized in IIT, PCT, and GWT, presenting them as complementary facets of a larger explanatory structure.

The notion of autonomy at the heart of IWMT also reflects a deeper lineage in biologically inspired theories of consciousness. In particular, Gerald Edelman's Theory of Neuronal Group Selection (TNGS), a theory grounded in Neural Darwinism, can be seen as an intellectual precursor to PCT. TNGS highlights how environmental interaction and selection shape neural development, emphasizing that the brain evolves not merely through internal mechanisms but in active exchange with the world (Edelman, 1987, 1993). While TNGS itself does not originate from phenomenological concerns, its stress on embodiment and ecological embeddedness resonates with many of the insights foregrounded in this paper.

Meanwhile, Bennett et al. (2024) propose an approach that aligns closely with phenomenological perspectives. Instead of assuming the existence of abstract mental states and thereby accepting a dualistic representational model that separates animal and environment, they conceive consciousness as a natural extension of biological life – that is, a phenomenon that emerges from the self-organization of embodied agents interacting continuously with their environment. Grounded in pancomputational enactivism—the view that the emergent properties of mind, as situated in the world, can be understood in computational or logical terms—their study reconstructs the emergence of consciousness through mathematical and logical formalism. This framework offers insight into how consciousness originates from physical embodiment and environmental engagement. Interpreted through the lens of phenomenology, their account reveals how more complex forms of consciousness, including higher-order stages, can be traced back to a primordial layer of pre-reflective self-awareness.

At the same time, their work draws conceptually from self-organization, a principle shared with IWMT's emphasis on autonomy, while also adopting the active inference framework at the heart of wide PCT. The central claim is that biological systems, unlike inert physical systems, are dissipative structures that maintain themselves by actively resisting entropy. This process of self-organization enables them to interpret sensory information hierarchically, based on internal needs and affective valence. Through this, organisms generate behavioral policies that differ according to the qualitative features of information processing – what the authors term “qualitative classifiers.” These processes are deeply embedded in natural selection, favoring systems that are better able to intervene in the world. Thus, the authors argue that subjective aspects of consciousness, such as feelings and motivations, are emergent properties of these adaptive, self-organizing systems.

Importantly, the study maintains that Phenomenal consciousness is grounded in the dynamics of self-organizing systems and originates from lower-level processes such as homeostatic regulation and basic sensation. These foundational systems form the experiential basis from which higher-order forms of consciousness, such as metacognition and reflective self-awareness, emerge. Accordingly, higher-order consciousness is not autonomous; it is contingent upon the existence of first-order selfhood. Without such foundational systems, an organism would lack not only reflective capacities but qualitative experience altogether. In short, metacognitive awareness alone is insufficient to account for Phenomenal consciousness. While posing a substantial challenge to HOT, this argument also points toward a potential reconciliation: one in which higher-order processes are not abandoned, but rather recontextualized as emerging from the embodied and inferential dynamics highlighted by PCT, offering a richer account of consciousness grounded in hierarchical processing.

Singhal and Srinivasan (2024) likewise advocate grounding scientific theories in phenomenological findings, focusing on consciousness as the lived sense of the present: the feeling of presence or temporal immediacy through which subjective experience unfolds in time. On this view, different theories of consciousness can be understood as capturing distinct aspects of how consciousness unfolds across time. Rather than seeking a single correct model, their approach reframes the debate: consciousness is not a static or unified entity but a temporally layered process, one that demands integration across multiple explanatory perspectives. Specifically, the authors propose a nested hierarchical paradigm inspired by temporal phenomenology (Singhal and Srinivasan, 2021). This framework suggests that consciousness operates simultaneously on multiple timescales, from fleeting sensory impressions to sustained episodes of awareness. It echoes Husserl's tripartite model of time-consciousness—primal impression, retention, and protention (Husserl, 1991, p. 28–35)—in proposing that each temporal layer corresponds to distinct cognitive functions, such as present-centered perception, memory of the immediate past, and anticipation of the near future. By integrating these layers, the model captures the dynamic, flowing nature of consciousness into a temporally extended field in which past, present, and future remain continuously linked. Grounding this view in phenomenological insight allows the framework to move beyond reductive accounts of consciousness, offering instead a richer and more temporally textured understanding of how different aspects of cognition contribute to the unified experience of the present moment.

To sum up, integrating multiple theories of consciousness is not an impossible task in principle. In fact, a more thorough and comprehensive understanding of conscious phenomena is likely to emerge when their respective explanations are considered from a broader perspective. Since each theory offers a distinct and legitimate account of the specific aspects it targets, identifying ways to fully leverage their explanatory strengths is essential, particularly if we are to approach *consciousness as a whole* as a viable subject of scientific inquiry.

## 6 Concluding remarks

The role of philosophy in empirical science is to delineate the object of inquiry with maximal conceptual clarity. It establishes the foundations of meaning and integrates them with empirical frameworks, ensuring that scientific methods are applied to the appropriate domain. This is especially vital in the study of consciousness, where the risk of misidentifying the explanandum is particularly high. Empirical theories of consciousness must remain accountable to phenomenological findings. Any theory that disregards the structures of lived experience cannot provide a valid account of conscious phenomena. The target of explanation must be *consciousness itself* , not merely *neural activity in the brain* described in representational terms. The essence of consciousness lies in subjectivity – the *immediate, lived sense of experience* that is given to us prior to abstraction. It is not reducible to qualia alone, nor is it captured solely through mechanisms of access. While transcending the Hard Problem remains the greatest challenge in the science of consciousness, it is important to recognize that qualia by themselves cannot account for the full structure of experience. Paradoxically, IIT, while committed to explaining qualia, ends up neglecting the functional architecture of conscious mental processes. This limitation is even more pronounced in more overtly reductionist theories such as GWT and HOT.

These limitations are not merely technical but point to a deeper conceptual fragmentation in how consciousness is approached across disciplines. If we are to move forward, what is needed is not just better models, but a more integrative perspective that remains attentive to the full range of conscious experience. *Rather than competing, the various empirical theories of consciousness must now move toward integration*. For instance, Phenomenal consciousness and Access consciousness are not mutually exclusive; they are interdependent dimensions of conscious phenomena. A unified framework that accounts for both is essential if we are to truly understand what consciousness is. I do not suggest that theoretical integration will miraculously resolve the enduring challenges of consciousness research. However, I hope this work contributes to shifting the discourse away from adversarial opposition and toward a more collaborative landscape, one in which the strengths of distinct theories can be brought into mutual alignment and constructive dialogue. While phenomenology may not be the ultimate key that unlocks all aspects of conscious mental properties (and reflective analysis reminds us that even the phenomenological attitude itself may at times be something to bracket!), we must be careful not to trivialize consciousness into mere operational constructs, nor allow our theoretical commitments to obscure what is most evident. Rather than fixating on a particular theoretical paradigm, we should, in the spirit of Husserl's call to “*return to the things themselves”*, begin from the phenomena of conscious life as they are given.

## Data Availability

The original contributions presented in the study are included in the article/supplementary material, further inquiries can be directed to the corresponding author.

## Footnotes

^1^It should be noted that analytic philosophy is not monolithic in its treatment of perception. Although representationalism has been widely discussed within the analytic tradition, as exemplified by Tye (1995) and Byrne (2001), it has been challenged by alternative theories, such as the naïve realism developed by Martin (2002) and the relationalism proposed by Brewer (2011), both of which hold that perception puts us in direct contact with the world. Even among representationalists, the idea that perception is “indirect” in the Cartesian sense is often explicitly rejected; see, for instance, Dretske (1995) and Tye (2000). Despite internal differences, representationalist theories generally share the assumption that perceptual experience is mediated by representational content.

^2^Denying the Hard Problem is essentially equivalent to denying the existence or the irreducibility of Phenomenal consciousness, as argued by Chalmers (1995). Strong reductionists and eliminativists, such as Churchland (1983) and Dennett (1991), attempt to sidestep the issue by rejecting the ontological reality of qualia, thereby rendering the Hard Problem moot by definition.

^3^It is helpful to distinguish between two strands within the phenomenological tradition that have challenged representationalism. The first, rooted in Husserl's transcendental idealism, treats consciousness as the *a priori* condition for the possibility of objectivity. The second, developed within the enactive and embodied approaches to cognition, criticizes representational metaphors by emphasizing the dynamic, sensorimotor coupling between organism and world. This paper is more aligned with the latter tradition, which seeks compatibility with empirical research without abandoning phenomenology's insights. At the same time, I would argue that the distinction between *a priori* universality and embodied situatedness in phenomenology need not be taken as a contradiction, at least within the framework of this paper. What this paper seeks to emphasize is that the essential structures of consciousness are always realized through historically and bodily situated acts of experience. This layered view preserves both transcendental and embodied-enactive insights within the phenomenological tradition.

^4^This sharply contrasts with third-person methodologies that seek to bypass or even eliminate the first-person perspective altogether, such as heterophenomenology. Heterophenomenology, proposed by Dennett, represents a methodological stance that stands in direct contrast to phenomenology. It advocates for a third-person approach to consciousness, arguing that subjective reports should be treated as behavioral data without committing to the existence of actual phenomenal states (Dennett, 1991, p. 76–77; Dennett, 2018). Dennett maintains that in order to remain scientifically neutral, researchers must abandon the first-person perspective in favor of what subjects say and do. This stands in opposition to phenomenology, which brackets naturalistic assumptions to uncover the structure of lived experience. While heterophenomenology claims objectivity through the exclusion of subjectivity, phenomenologists have critiqued this as a naïve stance. Since all scientific inquiry presupposes a conscious agent engaging in experience, the complete elimination of the first-person is neither feasible nor conceptually coherent (Jack and Roepstorff, 2002).

^5^Although Brentano's definition of intentionality remains foundational, it has also been taken up and developed in divergent ways within the phenomenological tradition. While Brentano emphasized intentionality as the mental directedness toward an object, later phenomenologists have challenged and expanded this interpretation. Gurwitsch (1979, p. 33) and Merleau-Ponty (2013, p. 338, 358) emphasize the field-structured and bodily-situated nature of intentionality, whereas Zahavi (2005) and Drummond (1990, p. 108-109, 113) stress its pre-reflective, structural mode of disclosure. Despite these differences, what unites these views is the understanding of intentionality not as a static relation between a subject and an object, but as the dynamic condition through which the world is experientially disclosed. This paper follows that broader orientation, treating intentionality as the structural mode of appearing rather than as mere object-directedness. This differs from analytic accounts that focus on propositional content or functional role; phenomenological intentionality is not about informational access to an object, but about the horizon-structured unfolding of meaning as disclosed through embodied subjectivity.

^6^Consequently, experiences such as affection and mood, which at first glance may seem non-intentional, can still be understood as intentional insofar as they express a mode of being situated in the world (Heidegger, 1996, p. 129; Sartre, 2021, p.450–452; Merleau-Ponty, 2013, p. 82).

^7^For a detailed discussion of this interpretation, see Zahavi (2005, p. 83–85, 87), who argues that both Heidegger's notion of *Dasein* and Sartre's account of pre-reflective self-consciousness affirm a minimal form of self-awareness as a structural feature of experience.

^8^However, some theories of consciousness, particularly Higher-Order approaches, challenge the idea that self-consciousness is necessary for phenomenality. Higher-Order theories are grounded in the transitivity principle, according to which a mental state is conscious only if it is represented by a higher-order state. Phenomenology, by contrast, begins with the lived givenness of experience, adhering to the “intransitivity principle”: that phenomenal consciousness is intrinsically self-revealing and does not require supplementary representation. What separates these perspectives is not merely theoretical preference, but a deeper divergence in how the relation between consciousness and pre-reflective/reflective self-awareness is understood at the level of first-person givenness. See Section 4 for further discussion.

^9^Husserlian transcendental phenomenology typically analyzes intentionality in terms of noetic–noematic correlation, structured by transcendental bracketing. Post-Husserlian and embodied approaches, on the other hand, do not reject this correlation but reinterpret it as a situated and affectively attuned mode of engagement. In this view, intentionality is not defined primarily by object-reference but by a subject's bodily, temporal, and intersubjective positioning within a meaningful world.

^10^Note that primal impression, retention, and protention are not merely psychological constructs but constitutive structures of temporal consciousness. According to this account, recollection is not a simple retrieval of past events, but a re-enactment within a temporal horizon already structured by retention and protention. The same applies to anticipation, which draws upon this dynamic temporal synthesis. These deeper structures form the ground from which higher-order temporal acts like memory and prediction emerge (Husserl, 1991, p. 41, 83, 118).

^11^While this section emphasizes selective attentional focus as a modality of agentive orientation, it remains important to clarify the relationship between attention and consciousness, especially given that attention selection is considered a key feature of subjectivity. Building on the idea that both attention and consciousness exist in multiple forms, Marchetti (2012) argues that non-focal or distributed attention may be constitutive of conscious experience, thereby challenging the view that the two are entirely separable.

^12^However, it should not be assumed that bodily actions regulated below the threshold of focal awareness are thereby non-intentional. Even when attention is not explicitly directed to them, such movements retain intentional structure insofar as they are non-reflexive and organized in accordance with practical goals (Merleau-Ponty, 2013, p. 144; Henry, 1975, p. 92).

^13^The terms “intentional matter” and “intentional quality” are used here in the Husserlian sense as developed in the Logical Investigations, rather than in their analytic usage. In this framework, intentional matter refers to the objective content toward which a conscious act is directed (e.g. “a glass of water”), while intentional quality refers to the particular mode of experiencing or intending that content (e.g. judging, desiring, doubting). These are both seen as essential components of the *act structure (Actstruktur)* and are analytically distinguishable but inseparable in lived experience (Husserl, 2001, Vol. II, 5th Investigation).

^14^However, note that there is considerable variation across Higher-Order approaches, depending on how they specify the mechanisms by which higher-order states generate consciousness. Accordingly, the strictness with which the Transitivity Principle is applied varies. Some hybrid theories, particularly those combining representationalism with more nuanced gradations of awareness, do acknowledge peripheral or preconscious states in ways that align partially with phenomenological intuitions (Gennaro, 2015; Kriegel, 2009; Van Gulick, 2004). This discussion, however, focuses on the general HOT framework rather than specific hybrid models.

^15^While not explicitly framed as phenomenological, Bennett et al.'s (2024) recent study supports a similar claim by demonstrating the precedence of self-consciousness in embodied, self-organizing systems. Their formal argument, developed through a pancomputational enactivist framework, contends that lower-order information processing must constitutionally precede higher-order representational states for consciousness to arise. For further discussion, see Section 5.

^16^The hierarchical distinction referenced here concerns levels within mental processing, not levels of neurobiological organization. It is important not to conflate thematic levels when discussing theories of consciousness. Phenomenology remains neutral on the issue of naturalizing the mind and is thus fully compatible with empirical findings, including hierarchical mechanisms in visual perception. This allows for theoretical compatibility. For instance, even if neural correlates of pre-reflective self-consciousness span multiple processing levels, this poses no contradiction within phenomenological logic. The same holds for Higher-Order theories: since HOT is not inherently reductionist, hierarchical levels in mental representation need not map directly onto neurobiological hierarchies.

^17^Note the shift in terminology from “phenomenological axioms” in version 3.0 of IIT (Tononi, 2012; Oizumi et al., 2014) to “phenomenal axioms” in version 4.0 (Albantakis et al., 2023). This change suggests a conceptual departure from the intrinsic properties of consciousness as traditionally understood in phenomenology. Similarly, the “ontological postulates” of version 3.0 have been renamed “physical postulates” in version 4.0. These shifts in language indicate a move away from a broadly ontological orientation toward a more narrowly physicalist framework, emphasizing the aim of realizing phenomenal axioms through physically instantiated correlates.

^18^For a more detailed critique of IIT's phenomenal axioms, see Bayne (2018), who argues that none of the five axioms are compelling from a phenomenological standpoint. While this paper does not pursue axiom-by-axiom analysis, Bayne's concerns reinforce the broader critique that IIT's conception of consciousness may be overly formal and detached from lived experience.

^19^Although the term “state consciousness” is not explicitly used in the PCT literature, Friston et al. (2018) discusses how attention and phenomenal content can both be explained through predictive coding: “This is potentially important, because it places attention in very close relation to the experience of prediction errors. … The inferential or sentient phenomenology is straightforward. In terms of a more phenomenological and quantitative experience, I think the story still holds. In other words, some form of attention is necessary to underwrite the access of ascending prediction errors to deeper levels of processing; such that they can revise our beliefs and expectations about states of the world” (Friston et al., 2018, p. 28).

^20^In the context of traditional psychological paradigms, GNWT also complements Signal Detection Theory (SDT), by offering a neurobiological framework that grounds SDT's behavioral observations. Several studies highlight the interdependence of these models (Van Vugt et al., 2018; King and Dehaene, 2014; Mashour et al., 2020). Like HOT, SDT presupposes a representationalist framework in which cognition involves the detection of signals by a subject distinct from the world. However, it does not directly address the phenomenological character of mental states such as hits or false alarms. Thus, SDT may coexist conceptually with HOT, just as GNWT can.
